# WFDC2 (HE4): A potential role in the innate immunity of the oral cavity and respiratory tract and the development of adenocarcinomas of the lung

**DOI:** 10.1186/1465-9921-7-61

**Published:** 2006-04-06

**Authors:** Lynne Bingle, Simon S Cross, Alec S High, William A Wallace, Doris Rassl, Guanglu Yuan, Ingegerd Hellstrom, Michael A Campos, Colin D Bingle

**Affiliations:** 1 Department of Oral Pathology, School of Clinical Dentistry, University of Sheffield, Sheffield, UK; 2 Academic Unit of Pathology, Division of Genomic Medicine, University of Sheffield Medical School, Sheffield, UK; 3 Diagnostic Services Department, Level 6 Medical & Dental School, University of Leeds, Leeds, UK; 4 Department of Pathology, University of Edinburgh, Edinburgh, UK; 5 Department of Pathology, Papworth Hospital, Cambridge, UK; 6 Academic Unit of Respiratory Medicine, Division of Genomic Medicine, University of Sheffield Medical School, Sheffield, UK; 7 University of Washington, Department of Pathology, Box 359939, Seattle, Washington, USA; 8 Division of Pulmonary and Critical Care Medicine, University of Miami, Miami, Florida, USA

## Abstract

**Background:**

The Whey Acidic Protein domain is an evolutionarily conserved motif found in a number of proteins, the best studied of which are antiproteinases involved in the innate immune defence of multiple epithelia. We recently characterised the WFDC2 gene which encodes a two WAP domain-containing protein, initially suggested as a marker for epididymis, and showed that it is highly expressed in the lung and salivary gland. The precise location of WFDC2 protein in these sites has not been described.

**Methods:**

We used immunohistochemistry to localise WFDC2 in normal tissues of the respiratory tract, naso- and oropharynx, as well as in chronically inflamed lung from Cystic Fibrosis and a range of pulmonary carcinomas. We have complemented these studies with molecular analysis of WFDC2 gene expression in primary human lung cell cultures.

**Results:**

WFDC2 is expressed in some epithelial cells of the upper airways as well as in mucous cells and ducts of submucosal glands. No staining was seen in peripheral lung. Intense staining is found in major salivary glands and in minor glands of the nose, sinuses, posterior tongue and tonsil. Studies with the related protein Secretory Leukocyte Protease Inhibitor (SLPI) show that although both proteins are expressed in similar tissues, the precise cellular localisation differs. Significant increases in expression and localisation of WFDC2 are seen in patients with Cystic Fibrosis. SLPI expression was greatly reduced in the same samples. In cultures of tracheobronchial epithelial cells, expression of WFDC2 and SLPI are differentially regulated during differentiation yet WFDC2 is not induced by pro-inflammatory mediators. The majority of adenocarcinomas stain with WFDC2 whilst a significant minority of squamous, small cell and large cell carcinomas exhibit focal staining. There is no clear association with tumour grade.

**Conclusion:**

We believe that these studies support the hypothesis that WFDC2 may be a component of the innate immune defences of the lung, nasal and oral cavities and suggest that WFDC2 functions in concert with related WAP domain containing proteins in epithelial host defence. We also suggest that WFDC2 re-expression in lung carcinomas may prove to be associated with tumour type and should be studied in further detail.

## Background

The WAP 4-disulphide core domain 2 (WFDC2) gene product, also known as human epididymis 4 (HE4), was originally identified as a transcript exclusively expressed in the epididymis and was proposed as a specific marker for this tissue [1]. WFDC2 is a member of the Whey Acidic Protein (WAP) domain family of proteins. The WAP domain is a conserved motif of approximately 50 amino acids, which contains eight cysteines found in a characteristic 4-disulphide core arrangement [2]. WAP domain proteins are usually small secretory proteins, which display a variety of functions [2,3]. Two of the best-studied members of this gene family are the antiproteinases, Secretory Leukocyte Protease Inhibitor (SLPI) and elafin. SLPI contains two WAP domains while elafin contains one. SLPI and elafin, protect against proteolytic enzymes released by inflammatory cells [3]. The major physiological function of SLPI and elafin is considered to be the inhibition of neutrophil elastase (NE), but they are also potent inhibitors of a variety of other proteinases [3]. Beside these shared antiproteinase activities, SLPI and elafin exhibit a range of other host defence functions [4]. For example, SLPI and elafin have antibacterial activities and both bind to bacterial lipopolysaccharide (LPS) [4-8]. SLPI has also been shown to block the *in vitro *replication of viruses [9] and to promote cutaneous wound healing in mice [10]. Furthermore, SLPI and elafin may have anti-inflammatory activities as SLPI inhibits monocyte/macrophage pro-inflammatory functions [11] and we have shown that elafin blocks inflammatory responses in the lungs of LPS treated mice [12]. Both SLPI and elafin therefore play a significant role in host defence.

On the basis of the similarity of WFDC2 to SLPI and elafin it was suggested that the protein may function as an antiproteinase within the male reproductive tract and may be important in the process of sperm maturation [1] although this function has never been proven. Indeed, although the function of WFDC2 remains unresolved a number of studies have reported that WFDC2 RNA is over-expressed in ovarian tumours [13-16] and in subgroups of lung adenocarcinomas [17,18]. Recent studies have also shown that WFDC2 protein is overexpressed in some types of ovarian cancer, principally serous and endometrioid tumours [16,19,20]. Such studies suggest that WFDC2 may play an undefined role in carcinogenesis and/or tumour progression. These studies also propose that WFDC2 may have some utility as a histological marker for ovarian cancer [16,19,20] and serum WFDC2 levels have been suggested to be a sensitive marker for ovarian cancer [19].

We recently showed that WFDC2 is expressed in a number of normal human tissues outside of the male reproductive system, including the trachea, lung and nasal epithelium and is also found in a subset of pulmonary epithelial-derived tumour cell lines [21]. The co-expression of WFDC2, SLPI and elafin in these tissues further suggests that WFDC2 may play an as yet undefined role in the host defence of the respiratory tract [21]. Disregulation of the proteinase/antiproteinase balance is thought to play a role in the development of many respiratory conditions including, chronic obstructive pulmonary disease (COPD) and cystic fibrosis (CF) [22]. As well as being altered in patients with a variety of lung diseases [23,24] it has been shown that both SLPI and elafin are regulated by proinflammatory stimuli [25,26]. Such observations suggest that WFDC2 may function to protect the lung in similar conditions and that expression of WFDC2 may be altered in pulmonary disease.

In this paper we have studied the distribution of WFDC2 throughout the normal respiratory tract and in oral and nasal tissues as well as in the lungs of patients with CF. Additionally we have studied the expression of WFDC2 in primary lung-derived epithelial cells. We believe that these results further support the hypothesis that WFDC2 may be involved in host defence and suggest that altered expression of this gene may influence host defence capabilities of the lung and may prolong pulmonary inflammation. We have also studied expression of WFDC2 in a range of pulmonary carcinomas and show that expression of the protein is associated with the majority of adenocarcinomas as well as in a significant minority of other lung tumour types.

## Methods

### Immunohistochemistry

For nasal, oral and lung tissue, serial sections were cut from formalin-fixed and paraffin-embedded tissue with appropriate ethical approval as described [27]. For normal tissues sections were taken so as to be as representative of normal architecture as possible although as all tissues were removed for medical reasons there was some evidence of disease in some sections. Sections from the major bronchi and peripheral lung were cut from 10 cases of normal lung and similar samples were also obtained from 10 patients with CF who were undergoing lung transplantation. We also purchased lung cancer tissue arrays from BioCat (Cat Number, US Biomax LC801 [28]), Superbiochips (Cat Number CCA-SBC [29]) and AccuMax (Cat Number A206 [30]). These arrays contained 150 lung cancer specimens of known diagnosis and stage, 10 metastatic samples from matched primary tumour cases, along with 15 samples of normal human peripheral lung tissues. The cores on these samples were 1.5–2 mm in diameter. The specific diagnosis was confirmed by one of us (W.A.W.). The slides were treated with 2% hydrogen peroxide in methanol to quench endogenous peroxidase. The following antibodies (and dilutions) were used in this study. A monoclonal antibody raised against human WFDC2 (clone 12H5) [18] (final dilution 1:500), a monoclonal antibody to human SLPI (clone 31, HBT, Holland, final dilution 1:250), a polyclonal antibody to human mucin (Muc5AC, a gift from David Thornton, University of Manchester, UK [31] final dilution 1:250) and SPLUNC1 [32] (final dilution 1:500). A standard antigen retrieval procedure was used for the SLPI and Muc5AC antibodies. Sections were incubated with 100% normal serum (horse for WFDC2 and SLPI and goat for Muc5AC and SPLUNC1) at room temperature for 30 minutes and then at 4°C overnight with the antibodies diluted as indicated above with normal serum. Rabbit or mouse IgG (DAKO) were used as negative controls on replicate slides. A Vectastain Elite ABC kit (Vector Laboratories) containing an appropriate biotin-labelled secondary antibody was used according to the manufacturer's instructions. Peroxidase enzymatic development was performed using a Vector NovaRed substrate kit resulting in red staining in positive cells. Sections were counterstained with haematoxylin, dehydrated to xylene and mounted in DPX. Staining of the lung cancer sections was scored as negative, focal or positive.

### Tissue culture

Human tracheobronchial epithelial (TBE) cell cultures were prepared by methods described previously [33]. Human tracheas and bronchi were obtained from lungs that were not deemed suitable for transplant through the Life Alliance Organ Recovery Agency of the University of Miami and approved by the local institutional review board. The mucosa from the larger airways was dissected and digested with protease, and released cells were collected by centrifugation. Cells were plated on collagen-coated culture dishes with bronchial epithelial growth medium [33] and harvested with trypsin after reaching confluence. These de-differentiated cells were plated onto 24-mm Transwell-clear culture inserts coated with human placental collagen. The cultures were maintained in a medium containing 50% DMEM and 50% LHC basal medium (Biosource International, Camarillo, CA, USA) supplemented with hormones and trace elements as described [33]. Upon reaching confluence (after 3–7 days), the medium from the apical surface was removed, leaving the top surface exposed to air (air-liquid interface cultures, ALI). To study the effect of retinoic acid (RA) on the expression of WAP protein genes normal [RA] (50 nm) was removed from the medium of fully differentiated cells after 14 days in culture.

Type II alveolar epithelial cells were isolated as previously described [34] from unused donor organ material supplied by the UK Human Tissue Bank with appropriate ethical approval. Cells were cultured in standard 12-well tissue culture plates. To study the effects of pro-inflammatory mediators on WAP protein expression in both primary cell types, cultures were stimulated with IL-1β, TNFα, (both from R & D Systems, Inc, Minneapolis, MN, USA) or *E.coli *LPS (Sigma, St Louis, MO, USA) for 24 or 48 hours prior to harvest. Stimulations for RNA analysis were performed on at least 3 occasions.

### RNA extraction and RT-PCR

Total RNA was isolated as previously described [35]. Reverse transcription was also performed as previously described [36] in a total volume of 20 μl using an oligo-dT primer and 1 μg of total RNA. PCR reactions were performed using 1 μl of each reaction product and the following primer pairs. WFDC2 F: 5' CGG CTT CAC CCT AGT CTC AG 3'; WFDC2 R: 5' AAA GGG AGA AGC TGT GGT CA 3'; Elafin F: 5' ACC TTC CTG ACA CCA TGA GG 3'; Elafin R: 5' GAT GAG AGA GGC AGC TCC AG 3'; SLPI F: 5' ATG AAG TCC AGC GGC CTC TT 3'; SLPI R: 5' CAT ATG GCA GGA ACT AAT CT 3'; Muc5B F: 5' ACT CCT GTC AAG TCC GCA TC 3'; Muc5B R: 5' ATC CAC GTG GGT GTA GGT GT 3'; CCSP F: 5' GTC ACA CTG GCT CTC TGC TG 3'; CCSP R: 5 GAG CAG TTG GGG ATC TTC AG 3'. All primer pairs were designed to cross introns. 27–35 cycles of the following program (94°C for 1', 60°C for 2' and 72°C for 3') generated the appropriately sized products, which were resolved on 2% TAE agarose gels, stained with ethidium bromide and photographed. Representative samples of each were directly cloned in TOPO pCRII (Invitrogen) and sequenced.

## Results

To study the localisation of human WFDC2 protein we used a monoclonal antibody raised against recombinant human WFDC2 [19]. This antibody has previously been used for both immunohistochemical analysis of WFDC2 in ovarian cancers as well as in ELISAs [19,37,38]. The antibody was also shown to specifically identify human WFDC2 in western blots of *in vitro *translation reactions (results not shown).

**Figure 1:**
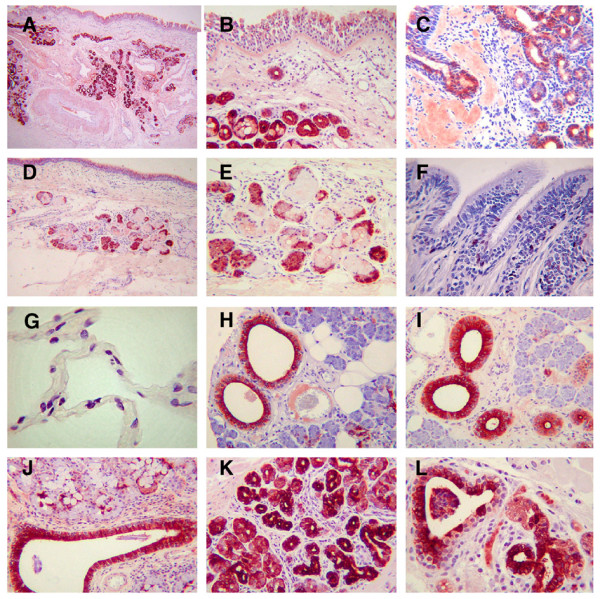
**Distribution of WFDC2 in the respiratory tract and salivary glands**. Immunohistochemistry for WFDC2 was performed as described in materials and methods. Sections show staining in nasal antral mucosa **(A, B)**, nasal polyps **(C)**, airway sub-mucosal glands **(D, E)**, epithelial cells of the airways **(F) **and negative staining in peripheral lung **(G)**. Staining was also found in parotid **(H)**, submandibular **(I) **and sub-lingual **(J) **glands and minor glands from the tongue **(K) **and tonsil **(L)**. The original magnification of the images was ×100 (A, B, C, D), ×200 (E, F, H, I, J) and ×400 (G, K, L).

In the light of our previous observation that significant expression of WFDC2 RNA was seen in tissue from the nasal passages [21], we initially stained sections from the maxillary sinus (antral) mucosa and nasal polyps. In these sections there was significant staining in the ductular epithelium of the minor glands, with less pronounced staining in the mucous cells of the glands themselves (Figure 1A, B). The surface epithelium of the nasal passages was also weakly positive. We stained nasal polyps from >15 individuals all of which demonstrated WFDC2 in ductular cells of the minor mucous glands (Figure 1C), although the intensity of staining varied and some regions of individual glands were found to be negative. The respiratory epithelium overlying the polyps was found to stain in a similar manner to the maxillary sinus. We then went on to examine pulmonary tissue and in sections from central bronchi the most prominent staining was again seen in sub-mucosal glands (Figure 1D, E) and was restricted to what phenotypically appear to be serous cells within the demilunes (Figure 1E). Cells within the surface bronchial epithelium from both major and minor airways were also stained (Figure 1D, F), although in the 10 samples studied both the intensity of staining and the number of cells stained varied (results not shown). In contrast to the positive staining seen in sections of the bronchi, none of the >20 samples of peripheral lung studied revealed clear WFDC2 staining in alveolar epithelium (Figure 1G).

In the light of our finding that WFDC2 staining was prominent in the minor glands of the upper airways, and our previous observation that WFDC2 was highly expressed in salivary gland RNA, we extended our studies to look at WFDC2 staining in both major and minor salivary glands. For all of these samples we stained sections from at least 3 individual patients. The three major salivary glands showed a striking distribution of WFDC2, predominantly in the ductular epithelium of the glands. In the parotid gland this intense staining of the excretory ducts was also accompanied by some staining in a few of the serous cells of the acini (Figure 1H). Similar staining in the excretory ducts was seen in the sub-mandibular gland along with staining in the striated and intercalated ducts, although in this tissue staining was not seen in serous cells within the acini themselves (Figure 1I). In the sublingual gland the strong ductular staining was also accompanied by staining within the serous demilunes of the predominantly mucinous acini, although the intensity of this staining was less marked than in the ducts. (Figure 1J). In samples of the posterior portion of tongue again the minor glands demonstrated strong WFDC2 staining (Figure 1K) and minor glands of the vallecular region of the tongue, associated with tonsil, also stained with WFDC2 (Figure 1L). In both of these glands the majority of the cells of the ducts expressed the protein very intensely. Most of the tonsil tissue, including tonsillar crypts or germinal centres, did not stain (results not shown).

These results clearly show that WFDC2 is present in a variety of glands associated with the upper respiratory tract, nasal passages and oral cavity and also suggest that the protein is present in a subset of upper airway epithelial cells. In addition, a significant site of WFDC2 protein localisation appears to be ductular cells of the major salivary glands. WFDC2 is not found at significant levels in the peripheral lung.

**Figure 2:**
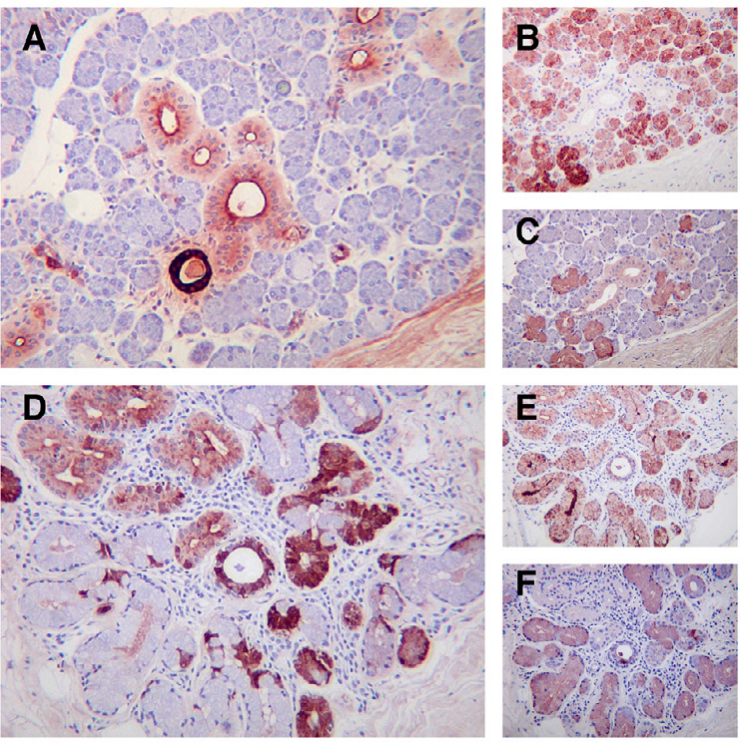
**WFDC2 and SLPI do not co-localise in major and minor glands**. The expression of WFDC2 **(A, D) **was directly compared with that of the related 2-WAP domain containing protein SLPI **(B, E) **and the mucous cell marker SPLUNC1 **(C, F) **in multiple glandular tissues as described in the materials and methods. Sections shown are submandibular gland **(A-C) **and bronchial sub-mucosal glands **(D-F)**. The original magnification of the images was ×200.

To complement our studies of the distribution of WFDC2, and to gain further insights into the cells that express the protein, we carried out more immunohistochemical studies, using additional antibodies on serial sections of multiple tissues. In the submandibular gland it is clear that expression of WFDC2 and the related 2-WAP domain containing protein, SLPI are almost mutually exclusive. As previously shown, (Figure 1I) WFDC2 was present in the ducts of the gland whereas SLPI was found in both the serous and (rarer) mucous cells of the gland (Figure 2A, B). SLPI staining was absent from the ducts. Staining for SPLUNC1 (a mucous cell marker [27]) was found to be strongest in the mucous cells of the gland with weak staining in the ductular cells (Figure 2C). A similar distribution pattern was seen in the submucosal glands of the bronchiolar epithelium where WFDC2 was found in the ductular cells and a subset of cells in the serous demilunes of the predominantly mucinous acini (Figure 2D). The majority of mucous cells did not stain with WFDC2. In this tissue, however, SLPI staining was found in some of the ductular tissues that express WFDC2 as well as the mucous cells of the gland (Figure 2E). SPLUNC1 was clearly localised in the mucous cells of the gland (Figure 2F). These results show that WFDC2 is distributed in a distinct subset of cells within both major and minor salivary glands but it is not completely co-expressed with the related protein SLPI.

**Figure 3:**
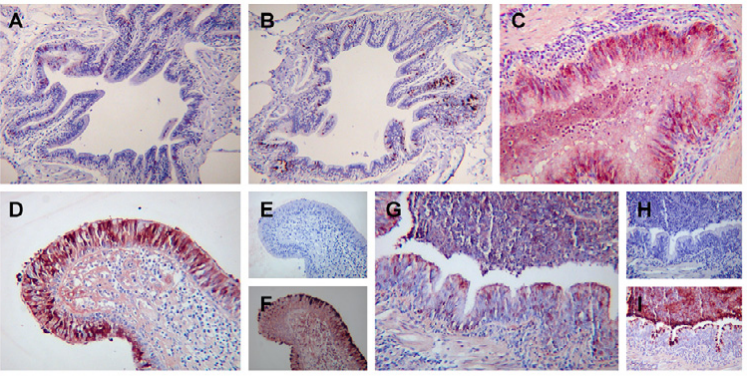
**WFDC2 is abnormally expressed in the Cystic Fibrosis lung**. Sections of normal **(A, B) **and Cystic Fibrosis lung **(C – I) **were stained with WFDC2 **(A, C, D, G)**, SLPI **(B, E, H) **and mucin 5AC **(F, I) **as described in materials and methods. The original magnification of the images was ×100 (A, B) and ×200 (C, D, E, F, G, H, I).

As chronic inflammation within the lung has been associated with alterations in the levels of SLPI and elafin [23-25], we studied WFDC2 expression in chronically inflamed lung using airway and peripheral lung sections from 10 patients who had undergone transplantation for CF. In sections of large airways from these samples there was no clear alteration in either the site or level of staining within the bronchiolar epithelial cells or the submucosal glands (results not shown). However, the situation in the smaller airways within the peripheral lung sections was markedly different. WFDC2 staining in the abnormal (hyperplastic) epithelium was found to be more generally distributed in the epithelium (Figure 3, C, D, G) compared to the situation seen in similar sized airways from CF disease-free lung (Figure 1F and Figure 3A). In addition to the greatly increased staining seen within the epithelium, the inflammatory mass within the airway lumen was also found to stain strongly for WFDC2 (Figure 3C and 3G). Sections stained with SLPI clearly did not display the same increased expression as was seen with WFDC2. In non-diseased small airways, SLPI was limited to cells with the phenotypic characteristics of goblet cells (Figure 3B). In CF sections stained with the same antibody, SLPI immuno-reactivity was markedly reduced compared to normal lung (Figure 3E, H) and was not detectable in the inflammatory exudates within the lumen of the small airways (Figure 3H). In contrast staining of sections with the goblet cell marker Muc5AC clearly showed that there was a significant increase in the number of mucous secreting cells in the CF airway (Figure 3F, I). The luminal content of these small airways also stained strongly for Muc5AC (Figure 3I). These results indicate that WFDC2 immunoreactivity is increased in the lungs of patients with CF and furthermore suggest the protein is secreted into the luminal contents of the diseased lung. This suggests that although expression of the WFDC2 gene may be mediated by inflammatory signals this may be different to that previously described for the related proteins SLPI and elafin.

**Figure 4:**
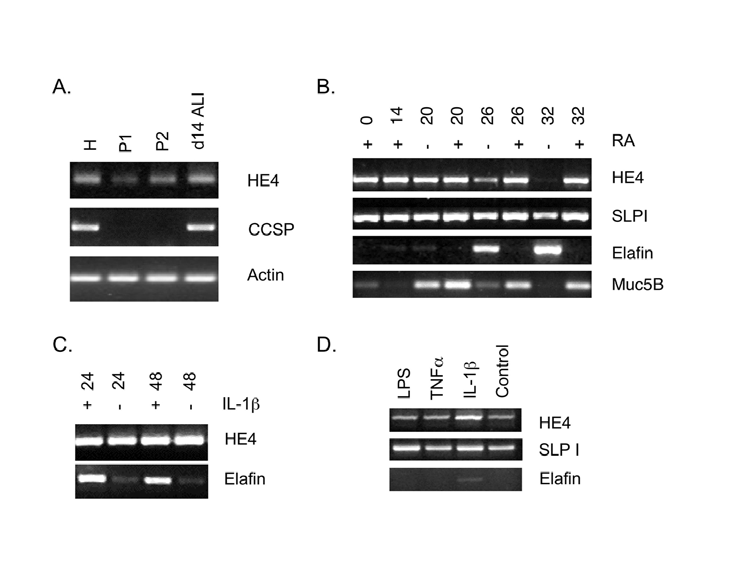
**WFDC2 is expressed in primary tracheal cells, differentiated tracheobronchial epithelial cells and alveolar Type II epithelial cells**. **A**. Expression of WFDC2 was studied by PCR using freshly harvested human tracheal cells (H) and samples taken from the cultures at two stages (P1, P2) as the cells were established at an air liquid interface (ALI). Expression of the non-ciliated tracheal cell marker CCSP was also monitored in the same samples. **B**. Expression of WFDC2 was studied by PCR in samples of primary tracheobronchial epithelial cells during retinoic acid depletion and the subsequent loss of the differentiated phenotype. The normal concentration of retinoic acid (50 nm RA) was removed (-) over the indicated period of days. RA withdrawal began at day 14. Expression of the related WAP domain containing proteins SLPI and elafin and the non-ciliated and mucous markers CCSP and Muc5B were also studied in the same samples. **C**. Expression of WFDC2 and elafin was studied by PCR in samples of differentiated primary tracheobronchial epithelial cells following treatment with IL-1β for 24 and 48 hours **D**. Expression of WFDC2, SLPI and elafin was studied by PCR in samples of primary alveolar epithelial pneumocytes (Type II cells) following treatment for 24 hours with LPS **(2)**, TNFα **(3) **or IL-1β **(4)**.

To directly address the question of potential regulation of WFDC2 by inflammatory mediators we performed a series of RT-PCR studies using primary human lung derived cells. In our previous study we had shown that WFDC2 was expressed in a number of lung cancer cell lines, some of which also expressed SLPI and elafin [21]. Given the localisation of WFDC2 staining in cells of the bronchiolar epithelium we initially chose to study primary cells from this region. We confirmed that WFDC2 mRNA was readily detectable in submandibular and parotid gland and in NCI-H358 cells (results not shown). When RT-PCR reactions were performed on bronchial epithelial cells directly harvested from healthy donor lungs WFDC2 mRNA was readily detected (Figure 4A, lane H). Reduced expression of WFDC2 was seen as the cells underwent the process of de-differentiation when plated in collagen-coated dishes for 3 days, allowed to expand (P1), and plated again for a second passage (P2). During this period of submerged culture the cells lose features of differentiation, confirmed by the complete loss of expression of the Clara cell secretory protein (CCSP) gene, a marker of differentiated bronchiolar cells (Figure 4A). When the cells were placed at an air-liquid interface they re-differentiated as evidenced by the visual appearance of cilia (results not shown) and re-expression of CCSP. At this stage, expression of WFDC2 was increased to levels similar to those observed in directly harvested cells (Figure 4A, lane d14). Similar results were found in two other sets of cells (results not shown).

To further investigate the role of differentiation of the ALI cells in influencing the expression of WFDC2 we modulated the differentiation status of the cells by removing retinoic acid from the culture medium. 50 nm RA is normally present in the culture medium of these cells and has been shown to be required for full differentiation of the cultures [39]. Removal of RA from these cultures led to a progressive loss of WFDC2 expression such that following 18 days of RA withdrawal, expression was essentially lost (Figure 4B). Levels of expression of SLPI in the same samples were clearly not influenced by the removal of RA (Figure 4B) whereas, in marked contrast, expression of elafin almost completely mirrored that of WFDC2 with the RA depleted cells having significantly more expression of the elafin gene. The requirement for RA to maintain the mucous secretory phenotype of the cells was shown by the loss of expression of Muc5AC that paralleled the loss of WFDC2 (Figure 4B). Again we found similar results in two further sets of experiments with cells from different donors (results not shown).

We also examined the role of pro-inflammatory stimuli on the expression of WFDC2 in the ALI cells. For these studies differentiated cells were exposed to levels of IL-Iβ, TNFα and *E. coli *LPS known to induce expression of a variety of responsive genes. Neither TNFα nor LPS was found to induce expression of WFDC2 under any conditions (results not shown). Similar negative findings were found with IL-1β (Fig 4C) but this cytokine does upregulate elafin gene expression. The expression of SLPI mRNA was not significantly altered by any of these treatments (results not shown). These results appear to suggest that although expression of WFDC2 in ALI TBE cells is influenced by differentiation status, classical pro-inflammatory mediators do not appear to regulate the gene.

We have previously shown that SLPI and elafin are constitutively expressed in primary type II alveolar epithelial cell cultures and that elafin expression is induced by IL-1β in these cells [26], thus we performed similar induction studies with WFDC2. WFDC2 gene expression was readily detected in control Type II cells (Figure 4D). Stimulation with the same pro-inflammatory mediators as was used for the ALI cells suggested that WFDC2 was slightly induced by IL-1β, but was not responsive to either TNFα or LPS (Figure 4D). Again, and consistent with our previous studies [26], elafin expression was induced by IL-1β but was not altered by either TNFα or LPS treatment in the Type II cells. SLPI was not altered by any of these stimulations. We have also found a similar lack of induction of WFDC2 gene expression in lung cancer cell lines treated with pro-inflammatory mediators (results not shown). These results suggest that WFDC2 expression is not responsive to pro-inflammatory mediators in primary human lung cells and that the increased staining seen in the CF lungs may be due to a phenotypic alteration of the cell population rather than a direct transcriptional effect on the WFDC2 gene.

**Figure 5:**
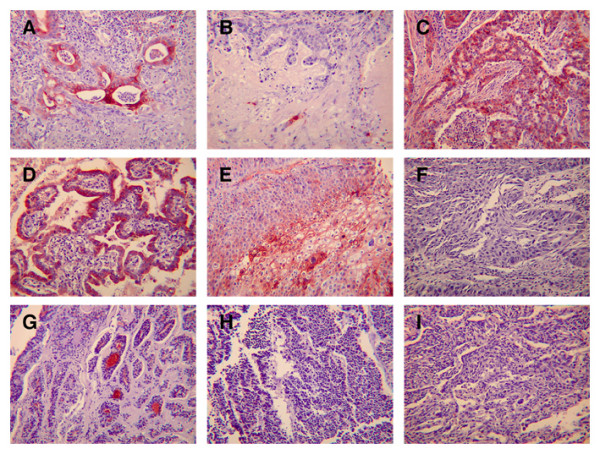
**Distribution of WFDC2 in Pulmonary Neoplasms**. Immunohistochemistry for WFDC2 was performed on three commercial lung cancer tissue arrays as described in materials and methods. Representative samples were chosen for imaging. Examples of focal **(A, B) **and positive staining **(C) **in adenocarcinomas, positive staining in a BAC **(D) **and focal staining in a case of squamous carcinoma **(E) **are shown as well as a negative case of squamous carcinoma **(F)**, focal staining in a case of adenoid cystic carcinoma **(G) **and a negative case of squamous carcinoma **(H) **and a mesothelioma **(I)**. The original magnification of the images was 200X.

In light of the multiple reports of disregulated WFDC2 expression in ovarian cancers [13-16] and the smaller number of reports that the gene may also be differentially expressed in sub-groups of pulmonary cancers [17,18] we stained sections of a variety of lung carcinomas with the WFDC2 antibody. To do this we used commercial tissue arrays that contained 150 individual cases from a range of different lung cancers. Representative results of this study are shown in Figure 5 and the data is summarised in Table 1. For representation of the data, each section was scored as being either negative, exhibiting focal positivity or being positive throughout the tumour. In focal positive cases the number and intensity of cells staining varied widely as did the intensity of staining in the positive cases. Due to the subjective nature of this assessment we have not attempted to sub-divide either of these two groups. It is clear from this data that both adenocarcinomas (Figure 5A–C) and bronchioloalveolar carcinomas (BAC, Figure 5D) exhibit the greatest percentage of positive staining tumours (>80%). In the majority of these strong, positive staining was identified throughout the tumour (Figure 5C and 5D), although focal positive staining ranging from a few cells within the tumour (Figure 5A) to focal staining of all of the abnormal ductular tissue (Figure 5B) was also seen. 12 of 63 cases of adenocarcinomas/BACs were negative. Focal staining was seen in 18 of 60 cases of squamous carcinoma (Figure 5E), the majority of these tumours were negative (Figure 5F). Focal staining was also seen in a single case of adenoid cystic carcinoma (Figure 5G) whilst 11 of 14 cases of small cell carcinoma (Figure 5H) and a single case of mesothelioma were negative (Figure 5I). The majority of cases of large cell carcinoma (8 of 11) were negative with the remainder exhibiting weak focal positive staining (results not shown). These results suggest that WFDC2 staining is predominantly associated with adenocarcinomas although a percentage of squamous, small cell and large cell carcinomas also exhibit focal positive staining.

**Table 1:** Summary of WFDC2 staining in lung tumours. Cores were designated as exhibiting no staining (Negative), focal positive staining (Focal) or as being positively stained (Positive).

	Negative	Focal	Positive
Squamous	41	18	1
Adenocarcinoma	12	14	31
BAC	1	3	2
Large Cell	8	3	0
Small Cell	11	2	1
Mesothelioma	1	0	0
Adenoid Cystic	0	1	0

## Discussion

We previously characterised the WFDC2 gene as part of an analysis of uncharacterised WFDC genes expressed in the airways [21]. Due to the similarities between WFDC2 and other WAP family members it has been suggested that the protein might function as an antiproteinase [1], although this function is still unproven. The WFDC2 gene was initially suggested to encode an epididymal specific protein [1], however, following the identification of a lung derived EST we were able to show that the WFDC2 gene is expressed in a range of tissues including the lung and salivary glands [21]. Other studies have identified WFDC2 over expression in ovarian and other cancers and have focussed on its potential use as a cancer marker [13-20,37,38]. The distribution and function of WFDC2 in normal tissues remains largely unresolved and thus we have extended our previous observations to study the localisation of WFDC2 in the oro- and nasopharynx and respiratory tract in an effort to gain a greater understanding of the function of the protein.

Our results clearly show that WFDC2 protein is readily detectable in multiple tissues from the oral cavity, nasopharynx and respiratory tract. These results are consistent with our previous studies of the gene [21]. Within these tissues the protein is most readily detected in the excretory ducts of both the major and minor glands as well as being expressed in a sub-population of airway epithelial cells. The expression of WFDC2 at these sites suggests that it may, like the related family members SLPI and elafin, be released into the secretions of the glands and the epithelium. In support of this suggestion WFDC2 has recently been identified as a component of bronchoalveolar lavage [40]. In the absence of significant WFDC2 protein in the secretory cells of the respiratory epithelium our results suggest that the protein enters the airway surface lining fluid via secretion from the sub-mucosal glands. We also directly compared localisation of WFDC2 with SLPI, a 2-WAP domain containing antiproteinase family member. SLPI is known to play a role in host defence in these regions and has previously been shown to be highly expressed in the submucosal glands [41]. Our results showed that although both proteins are present within the same tissues the precise cells in which they are found is not completely identical, indeed they are found in an almost mutually exclusive expression pattern in some tissues. We believe this observation suggests that the two proteins are under different regulatory control; this may further imply the functions of the proteins are quite distinct. As outlined in the introduction, an antiproteinase function for WFDC2 has been suggested but not demonstrated. The physical spacing of two of the conserved cysteine residues in the WAP domains of WFDC2 may, in fact, not allow sufficient space for the generation of an active antiproteinase site in the protein [2,3]. This would, however, not exclude a host defence role for WFDC2, as the host defence functions of other WAP domain containing proteins do not require an active antiproteinase domain [2-4].

A variation in intensity of staining was seen within samples and between samples suggesting WFDC2 may be locally produced in response to stimulation. We were unable to show induction of WFDC2 gene expression in ALI TBE cells but it appeared to be induced by IL-1β in primary type II cells. This suggests that expression of the WFDC2 gene is differentially regulated in different cell types although these finding need to be confirmed with a greater range of treatments and doses, perhaps using more sensitive techniques. In comparison, expression of elafin is only seen in type II epithelial cells after stimulation with IL-1β [26] whilst SLPI is constitutively expressed and not affected by cytokines. The fact that WFDC2, SLPI and elafin are differentially regulated by inflammatory stimuli suggests subtle differences in either their function or sites of action. The lack of induction by such stimuli does not exclude a host defence function for WFDC2 as not all host defence proteins are induced in this manner. For example SLPI is known to have potent anti-microbial activities, both anti-viral and anti-bacterial, but gene expression is not upregulated in our LPS stimulation studies. This observation is consistent with other reports [42,43]. Further studies of both gene and protein expression following treatment with a range of microbial products should clarify the function of WFDC2.

When we examined WFDC2 protein expression in the chronically inflamed lung from patients with CF we found no marked difference in the upper airways compared to normal samples but dramatically increased expression in the peripheral lung. In direct contrast, the expression of SLPI was dramatically reduced in the CF tissues; indeed it was very difficult to find any positive staining in the periphery of these damaged lungs. The tissue structure was completely altered in the peripheral lung with hyperplasia and many airways being obstructed with inflammatory exudates. Our PCR studies indicate that the increased expression of WFDC2 may not be a result of the action of inflammatory mediators but may be due to an increase in the number of cells secreting WFDC2 or a change in cell phenotype. Similarly, the loss of SLPI staining in the CF lung may also be due to phenotypic alteration in the epithelium. This suggestion is supported by the difference in expression of the WFDC2, SLPI and elafin genes during the de-differentiation/re-differentiation of the TBE cells. The alteration in expression of WFDC2 and SLPI proteins in the CF tissue is not likely to be the direct result of the CF mutation as it has recently been shown that TBE cells from normal and CF donors have remarkably similar gene expression signatures [44]. Further studies are required to elucidate the mechanism of this difference in CF. Potentially; levels of WFDC2 in CF lung secretions may influence the development of lung disease in this condition.

A number of different expression studies have clearly identified that WFDC2 is disregulated at the RNA level in ovarian cancers [13-16] and a recent study has suggested that measuring levels of WFDC2 in the serum of patients with ovarian cancer may be useful in monitoring disease progression [19]. Furthermore immunohistochemical studies, coupled with molecular classification array experiments, have shown that re-expression of WFDC2 may be associated with specific types of ovarian cancers [16,20]. The association of WFDC2 re-expression with other types of cancers has not been addressed in any great detail, although it has been shown in molecular classification studies that WFDC2 expression is associated with different subtypes of adenocarcinomas and some other lung cancer types [17,18]. Our present results clearly show that WFDC2 protein is readily detectable in a range of lung tumour types. Principle among these are adenocarcinomas, where the majority of cases were found to be positive for the protein. This study also clearly shows that WFDC2 expression is not purely associated with this tumour type as a significant percentage (20–30%) of other tumour types, for example squamous, small and large cell carcinomas, also exhibited focal positive staining for the protein. Although we saw clear expression of WFDC2 in the majority of cases of adenocarcinomas, there were also a number of cases (21%) that were negative. We were unable to make a clear association between the type of staining pattern and tumour grade in the group of samples that we have examined thus far, but it is clear that they must express a different subset of secreted proteins. Such differential expression of putative marker genes has been shown to yield distinct "molecular signatures" in lung carcinomas [17], which may be useful in the development of specific diagnostic and prognostic markers. Indeed two studies have shown that pulmonary adenocarcinomas can be classified into different molecular phenotypes where classification is associated with prognosis [17,18]. In these studies low expression of WFDC2, as judged by gene chip analysis, was associated with poor outcome [17,18]. One of these studies also showed that some groups of non-adenocarcinoma lung tumours express increased WFDC2 RNA levels as measured by chip analysis [17]. It is tempting to speculate that the immunohistochemical study that we report here may perhaps reflect the different sub-classifications of lung tumours identified in these expression array papers. Such a hypothesis would require us to extend this analysis into a significantly larger series of cases with better survival data and case histories. The reason for the increased expression of WFDC2 in some of the other pulmonary malignancies is also not clear and will require further studies.

## Conclusion

We have been able to show for the first time the precise expression pattern of WFDC2 in the respiratory tract. The protein, though found in a subset of epithelial cells and present in the ASL fluid is predominantly present in the mucous cells of the sub-mucosal glands of the upper airways as well as in minor glands in the nose, sinuses, posterior tongue and tonsil and the ducts of the major salivary glands. These locations are all sites of production of host defence proteins and the presence of protein in the glandular and epithelial secretions supports the suggestion that WFDC2 is involved in host defence. We have also shown that WFDC2 is expressed in the majority of cases of adenocarcinoma of the lung as well as being found in a significant number of squamous, small cell and large cell carcinomas of the lung suggesting it may have some diagnostic and/or prognostic value. However, further studies are needed to determine the precise function and regulation of WFDC2 expression.

## Competing interests

The author(s) declare that they have no competing interests.

## Authors' contributions

LB: participated in the design and coordination of the study, carried out all of the immunohistochemical studies and co-authored the draft of the manuscript.

SSC: provided invaluable histology expertise and contributed to the manuscript

ASH: provided all of the oral and oral tissues, analysed the immunohistochemistry of these tissues and contributed to the manuscript.

WAW: provided the normal lung tissues, analysed the immunohistochemistry of these tissues, confirmed the diagnosis of the lung array samples and contributed to the manuscript.

DR: provides the cystic fibrosis tissues, analysed the immunohistochemistry of these tissues and contributed to the manuscript.

GY: carried out all of the RT-PCR studies.

IH: provided the WFDC2 monoclonal antibody and contributed to the manuscript.

MAC: facilitated the culture of the tracheobronchial cells and contributed to the manuscript.

CDB: conceived of the study, participated in the design and coordination of the study and co-authored the draft of the manuscript
